# Cerebrovascular expression of proteins related to inflammation, oxidative stress and neurotoxicity is altered with aging

**DOI:** 10.1186/1742-2094-7-63

**Published:** 2010-10-11

**Authors:** Debjani Tripathy, Xiangling Yin, Alma Sanchez, Jinhua Luo, Joseph Martinez, Paula Grammas

**Affiliations:** 1 Garrison Institute on Aging, Texas Tech University Health Sciences Center, Lubbock, Texas

## Abstract

**Background:**

Most neurodegenerative diseases are age-related disorders; however, how aging predisposes the brain to disease has not been adequately addressed. The objective of this study is to determine whether expression of proteins in the cerebromicrovasculature related to inflammation, oxidative stress and neurotoxicity is altered with aging.

**Methods:**

Brain microvessels are isolated from Fischer 344 rats at 6, 12, 18 and 24 months of age. Levels of interleukin (IL)-1β and IL-6 RNA are determined by RT-PCR and release of cytokines into the media by ELISA. Vessel conditioned media are also screened by ELISA for IL-1α, monocyte chemoattractant protein-1 (MCP-1), tumor necrosis factor-α, (TNFα), and interferon γ (IFNγ). Immunofluorescent analysis of brain sections for IL-1β and IL-6 is performed.

**Results:**

Expression of IL-1β and IL-6, both at RNA and protein levels, significantly (p < 0.01) decreases with age. Levels of MCP-1, TNFα, IL-1α, and IFNγ are significantly (p < 0.05-0.01) lower in 24 month old rats compared to 6 month old animals. Immunofluorescent analysis of brain vessels also shows a decline in IL-1β and IL-6 in aged rats. An increase in oxidative stress, assessed by increased carbonyl formation, as well as a decrease in the antioxidant protein manganese superoxide dismutase (MnSOD) is evident in vessels of aged animals. Finally, addition of microvessel conditioned media from aged rats to neuronal cultures evokes significant (p < 0.001) neurotoxicity.

**Conclusions:**

These data demonstrate that cerebrovascular expression of proteins related to inflammation, oxidative stress and neurotoxicity is altered with aging and suggest that the microvasculature may contribute to functional changes in the aging brain.

## Background

Diseases of the CNS are for the most part age-associated disorders. A direct relationship exists between aging and increasing incidence of neurodegenerative disease [1]. In age-associated diseases inflammation and oxidative damage are important features of brain pathology and are often found together [2]. Despite a large body of data linking inflammation and oxidative stress to the pathology in age-related diseases, there is insufficient knowledge about the effects of aging alone, in the absence of disease, on inflammatory mediators and oxidative stress in the brain.

An age-related increase in plasma and circulating levels of tumor necrosis factor - alpha (TNFα), interleukin-1 beta (IL-1β), interleukin-6 (IL-6), tumor necrosis factor receptor (TNFRs) and interleukin-1 receptor antagonist (IL-1RA) [3-6] has been shown in some studies, while other investigations show no age-related increases in TNFα or IL-6 [7,8]. Similarly, an increase in IL-6 is reported in a randomly selected population of elderly Americans (70 and above) but not in a population identified as "strictly healthy" [7]. Twenty-four hour lipopolysaccharide (LPS) stimulation of whole blood supernatants finds lower levels of TNFα and IL-1β but not IL-6 in samples derived from elderly compared to young control samples [9]. Animal studies comparing young and aged rodents show a decrease in chemokine expression in a dermal injury model but an increase in pro-inflammatory cytokine expression in coronary arteries in aged animals [10,11]. In addition, there is disparity between cellular responsiveness and circulating plasma levels. There is an increase in serum kinin levels with aging but aortic endothelial cells from old Fischer 344 rats are hyporesponsive to exogenous bradykinin [12]. In human studies, IL-6, but not IL-1β and TNFα production by peripheral mononuclear cells is increased in aged subjects compared to young cohorts [13]. The effect of aging on the expression of cytokines and chemokines is controversial and inconsistent. In part, these discrepancies could reflect heterogeneity among different tissues examined. There is an increase in pro-inflamatory cytokine expression, detected by microarray, in coronary vessels of 25 month old Fischer 344 rats compared to vessels of young rats [10]. In contrast, a study examining the myocardial response to infarction in senescent mice shows a decrease in neutrophil and macrophage infiltration and reduced cytokine and chemokine expression in myocardial tissues compared to young mice [14].

Oxidative stress, which results from an imbalance between reactive oxygen species generation and antioxidant enzyme capacity, is particularly important for the brain because of its high metabolic rate. A number of indices of oxidative stress such as protein oxidation, lipid peroxidation, DNA oxidation and 3-nitrotyrosine formation as well as diminished levels of antioxidants such as superoxide dismutase (SOD) have been documented in neurodegenerative diseases [15]. In aging an increase in the amount of oxidized proteins has been reported, as measured by the level of intracellular protein carbonyls or nitrotyrosine [16]. Evidence from a number of studies shows aging-associated accumulation of oxidatively damaged DNA in the brain and other organs with limited cell proliferation [17].

The brain microvasculature appears to be a site of convergence for inflammatory and oxidative processes in neurodegenerative disease. Isolated brain microvessels obtained from Alzheimer's disease (AD) patients have high levels of both cell-associated and soluble cytokines and chemokines including IL-1β, IL-6, IL-8, TNFα, transforming growth factor-β (TGF-β) and monocyte chemoattractant protein-1 (MCP-1) compared to age-matched non-AD controls [18-20]. Also, in AD a damaged microcirculation releases high levels of the reactive oxygen species nitric oxide [21]. Finally, we document that in AD dysfunctional microvessels release factors that are directly injurious to neurons in culture [22]. Although age is the dominant risk factor for the development of neurodegenerative disease, the effect of aging on inflammatory, oxidative and neurotoxic processes in the brain microvasculature has not been explored. The possibility that the cerebromicrovasculature is functionally altered in aging is suggested by data showing that microvessels or vessel-conditioned media from elderly non-demented patients, although significantly less lethal than AD-derived vessels, injures neurons *in vitro *[22].

The objective of the current study is to determine whether expression of proteins in the cerebromicrovasculature related to inflammation, oxidative stress or neurotoxicity are altered in an animal model of aging.

## Materials and methods

### Microvessel isolation and preparation of conditioned media

Animal procedures were performed in accordance with NIH "Guide for the Care and Use of Laboratory Animals" and Texas Tech University Health Sciences Center Institutional Animal Care and Use Committee (IACUC) guidelines. Male Fisher rats 344 6, 12, 18 and 24 month old were purchased from NIA. The rats weighed approximately 450 to 500 g. Upon delivery they were housed in individual ventilated cages with bedding and free access to water for a week prior to microvessel isolation. Each age group consists of 24 male Fisher 344 rats and the microvessel isolation performed in triplicate [23]. Cerebral cortices were homogenized in cold Hank's balanced salt solution (HBSS) without calcium and the homogenate centrifuged at 3000 g for 15 min at 4°C. The pellet was resuspended in cold HBSS containing 15% dextran and 5% fetal bovine serum (FBS) and centrifuged at 4500 g for 20 min at 4°C. The supernatant discarded and the pellet filtered through a 150 μm sieve and microvessels collected on a 53 μm sieve. Microvessels were resupended in Dulbecco's modified Eagle's medium (DMEM) containing 10% FBS and dimethyl sulfoxide and stored frozen in liquid nitrogen until use. The purity of the microvessel preparations was assessed by phase contrast microscopy.

Microvessels from 6,12,18 and 24 month old rats were thawed and centrifuged at 2000 g for 15 min. Microvessels (50 μg/sample) were washed three times with cold HBSS and resuspended in 500 μl serum-free DMEM containing 1% lactalbumin hydrolysate (LAH) for 8 h at 37°C in a 95% CO_2_/5% O_2 _incubator and then centrifuged (2000 g). Supernatants were collected for neurotoxicity analysis and determination of inflammatory proteins.

### Cerebral cortical cell culture and measurement of neuronal cell death

Rat cerebral cortical cell cultures were prepared as previously described [22,24]. Cortices were isolated from 17-day gestation rat fetuses, washed with HBSS, triturated in 10 ml Brooks-Logan solution containing 0.025% trypsin and centrifuged for 10 min at 800 g. The supernatant was discarded and the pellet was triturated and filtered through a 210 μm mesh. The cells were plated at a density of 500,000/ml in media containing DMEM supplemented with 2 mM

L-glutamine, 10% heat-inactivated horse serum, and antibiotic/antimycotic on 6-well plates coated with poly-L-lysine. Experimental treatments were carried out on day 8 in Neurobasal medium containing N-2 supplement, 0.5 mM L-glutamine and antibiotic/antimycotic (treatment medium).

For the neurotoxicity assay, cerebral cortical cell cultures were incubated with 100 μl conditioned media for 24 h. Neuronal cells were washed with phosphate buffered saline (PBS) and incubated with the MTT reagent 3-(4, 5-dimethylthiazol-2-yl)-2-5-diphenyl tetrazolium bromide reagent (1:40 dilution) for 5-10 min at 37°C. The formazon product, which is produced only by live cells, was quantified by colorimetric assay at 490 nm (Cell Titer 95 Aqueous solution cell proliferation assay, Promega, Madison, WI). In each experiment, the number of control cells *i.e*. viable cells not exposed to any treatment was defined as 100%.

### Measurement of protein carbonyl content

Protein carbonyl content in microvessels was determined using the OxyBlot protein oxidation detection kit (Chemicon, Temecula, CA). Proteins extracted from brain microvessels from 6, 12, 18 and 24 month old rats were quantified using Bradford reagent. Equal amounts of protein (5 μl) were denatured by adding 5 μl of 12% SDS. The proteins were derivatized by adding 10 μl of 2, 4 dinitrophenylhydrazine (DNPH). The derivatization control solution without DNPH served as negative control. The samples were incubated at room temperature for 15 min and neutralized with equal amounts of neutralization solution. Both the treated sample and the negative control were subjected to dot blot analysis. Oxidized proteins were detected with anti-DNPH antibodies provided in the kit. Band intensities were quantified using the Quanitity One software (Bio-Rad) and represented as relative units.

### Detection of inflammatory proteins by ELISA and RT-PCR

IL-6 and IL-1β released into the supernatant were detected with rat custom 9 plex SL4638 Search Light sample testing service (Pierce Biotechnology, MA). To confirm the testing service results ELISA measurements were performed for IL-1β using ELISA kit RLB00 (R&D Systems, MN), according to manufacturer's instructions. Monocyte chemotactic protein-1 (MCP-1), TNFα, interferon γ (IFNγ) and IL-1α were also determined by the rat custom 9 plex SL4638 Search Light sample testing service.

Total RNA was isolated from rat brain microvessels with Trizol reagent (Invitrogen, Carlsbad, CA) and 1 μg of RNA was reverse transcribed using oligo dT primers (Roche Applied Science) and amplified by PCR. PCR amplification was performed with cDNA, gene specific primers (Table 1) as follows: denaturation for 2 min at 95°C, 30 cycles of 95°C for 40 sec, 55°C for 40 sec and 72°C for 2 min and a final extension at 72°C for 5 min. The PCR products were visualized in a 1.5% agarose gel containing 0.5 μg/ml ethidium bromide under UV trans-illumination light with a gel documentation system (Quantity One analysis software, Bio-Rad).

**Table 1:** Primers used for RT-PCR

Gene	Orientation	Sequence	Amplicon Size (bp)
IL-1β	Left primer	AGCAGCTTTCGACAGTGAGGAGAA	182
	Right primer	TCTCCACAGCCACAATGAGTGACA	

IL-6	Left primer	CCGGAGAGGAGACTTCACAG	428
	Right primer	GAGCATTGGAAGTTGGGGTA	

GAPDH	Left primer	TCTGCATCTGGCAAAGTGGAGACT	101
	Right primer	TTGAACTTGCCGTGGGTAGAGTCA	

### Western Blot determination of MnSOD

Total protein was extracted from microvessels with lysis buffer containing 0.1% SDS, 1% Triton X-100 and 0.5% phenylmethyl sulfonylfluoride. Protein estimation was performed with Bradford reagent (Bio-Rad). Equal amounts of protein were run on a 12% polyacrylamide gel, transferred on to a PVDF membrane, blocked with 5% milk solution (non-fat dry milk in Tris-buffered saline Tween-20) and immuno-blotted with MnSOD (ab 16956,1:1000) and GAPDH (MAB374, 1:1000) primary antibodies for 2 h. Membranes were washed with Tris-buffered saline Tween-20 and incubated for 1 h with peroxidase- conjugated secondary antibodies. After extensive washing to remove unbound antibodies, membranes were developed with chemiluminescence reagents. Band intensities were quantitified using Quantity One software (Bio-Rad) and graphically expressed as intensity units which reflects the average intensity over the area of the band normalized with that of GAPDH.

### Immunofluorescence staining of brain tissue sections

Immunofluorescence labeling of rat brain tissue sections was performed as described previously [25]. Briefly paraffin sections (5 μm) were deparaffinized and rehydrated in ethyl alcohol (100% to 70%). After heat-induced epitope antigen retrieval, sections were washed in Tris-buffered saline with Tween (TBST), blocked with 10% donkey serum at room temperature for 2 h and sections incubated at 4°C overnight with primary antibodies against: IL-1β (1:50, sc-7884, Santa Cruz Biotechnology, CA), IL-6 (1:50, sc-1265-R, Santa Cruz Biotechnology, CA), MnSOD (1:200, ab16956, Abcam) or Von Willebrand Factor (VWF) (1:50, sc-8068, Santa Cruz Biotechnology, CA) in TBS containing 2.5% donkey serum. Following three washes with TBST, sections were incubated with FITC conjugated secondary antibodies (1:400, Invitrogen, Carlsbad, CA) at room temperature for 1 h. All sections were counter stained with nuclear marker DAPI (blue). Fluorescent images were captured using an Olympus IX71 microscope and quantified with HAMAMATSU imaging software.

### Statistical analysis

Data from each experiment were expressed as mean ± standard deviation (SD). A one-way ANOVA followed by Bonferroni's multiple comparison tests for multiple samples were performed. Statistical significance was determined at p < 0.05.

## Results

### Microvessel-conditioned media from aged rats caused neuronal cell death

**Figure 1:**
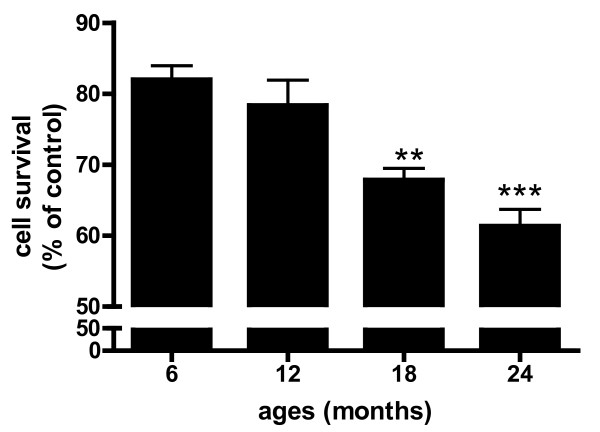
**Microvessels isolated from 6, 12, 18 and 24 month old rats were resuspended in serum-free media for 8 h, equal volumes of conditioned medium (50 μl) was added to cerebral cortical cultures and incubated for 24 h at 37°C**. Cell viability was determined by MTT assay. Viable cells not exposed to conditioned medium were defined as 100%. Results are mean ± SD from 2 separate experiments performed in triplicate. **p < 0.01 vs. 6 months; ***p < 0.001 vs. 6 months.

Based on our previous work showing that microvessels isolated from the brains of AD patients release neurotoxic factors [22], we examined the ability of isolated brain microvessels to cause neuronal cell death as a function of age. Microvessels isolated from Fischer 344 rats of 6, 12, 18, and 24 months of age were incubated in serum free media for 8 h and the conditioned media added to primary cortical cultures for 24 h. The results showed that with increasing age conditioned media from brain vessels evoked neuronal cell death. The ability of vessels to cause neuronal cell death was significant (p < 0.01) at 18 months of age (Figure 1).

### Aging increased oxidative stress and decreased antioxidants in brain microvasculature

**Figure 2:**
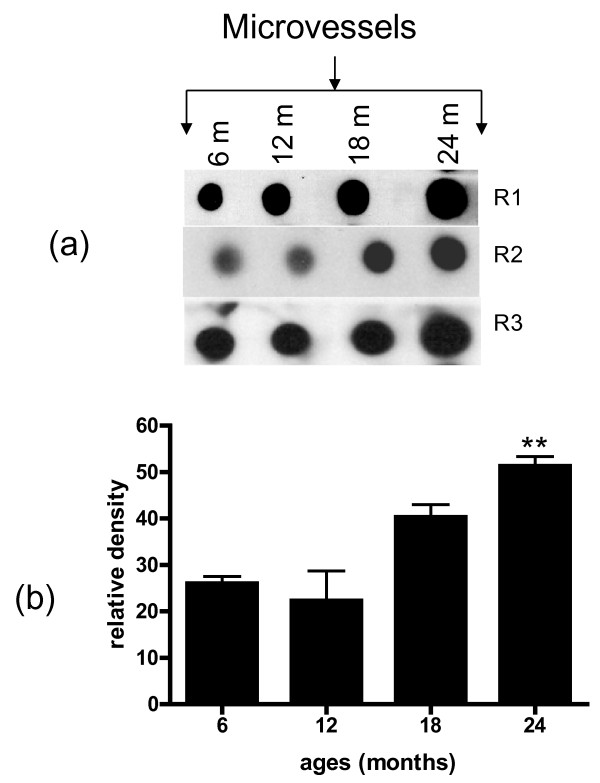
**Protein lysates from microvessels of 6, 12, 18 and 24 month old rats were derivatized with diphenyhydrazine (DNPH) and carbonyl formation identified with anti-DNPH**. Dot blot assay from 3 experiments (R1-R3) is shown in Figure 2a. Densitometric scan of dot blots (2b) is representative of 3 experiments performed in triplicate. **p < 0.01 vs. 6 months.

The oxidative modification of proteins by reactive oxygen species can be quantitated by measurement of protein carbonyl content. In the current study protein lysates from brain microvessels isolated from 6, 12, 18 and 24 month old rats were derivatized with 2, 4 dinitrophenyl hydrazine. Dot blot analysis using an antibody to DNPH showed an increase in vascular oxidative stress with age (Figure 2a). Densitometric scan of the data demonstrated that this increase was significant (p < 0.01) in animals 24 months of age (Figure 2b).

**Figure 3:**
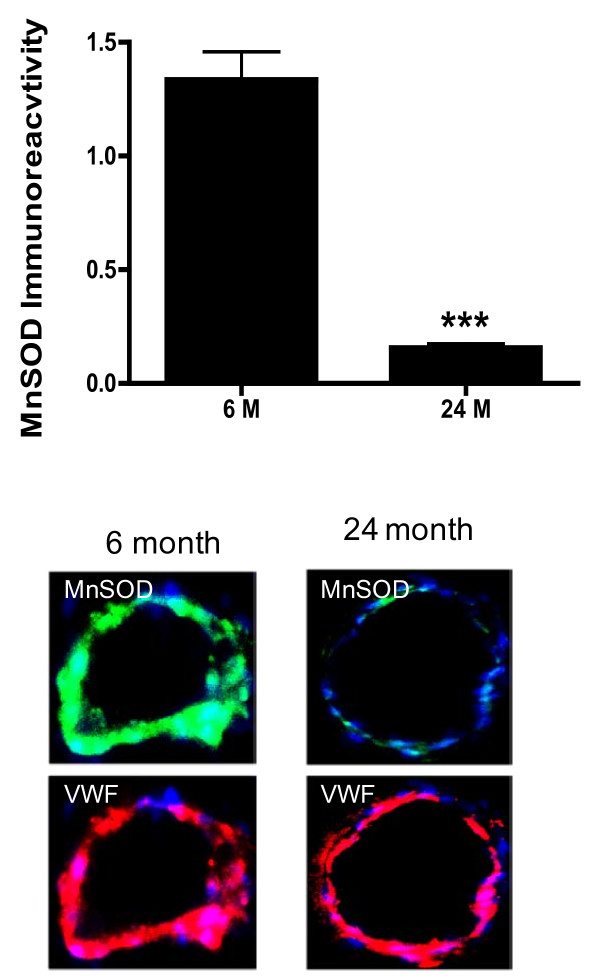
**Brain tissue sections from 6 and 24 month old Fisher 344 rats were stained for nuclear stain DAPI (blue) and either MnSOD (green) or the endothelial cell specific marker VWF (red)**. Quantitative comparison of MnSOD expression between vessels derived from 6 month and 24 month old animals is shown in bar graph. Data are mean ± SD (n = 6). ×20. ***p < 0.001 vs. 6 months.

**Figure 4:**
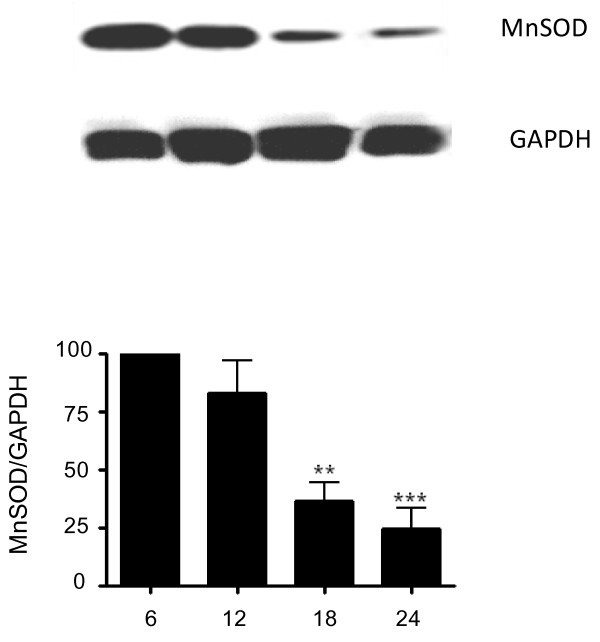
**Total protein extracted from brain microvessels obtained from 6, 12, 18 and 24 rats was resolved with 12% SDS-PAGE gel**. MnSOD and GAPDH were detected with specific antibodies. Lower panel shows the relative intensity of bands of MnSOD to GAPDH. Data are mean ± SD from 3 experiments. **p < 0.01 vs. 6 months; ***p < 0.001 vs 6 months.

Because increased oxidative stress could reflect increased radical generation, diminished antioxidant capacity or both, we explored the vascular expression of the antioxidant protein MnSOD in brain sections of rats at 6 and 24 months of age. Immunofluorescent results showed that reactivity to the MnSOD antibody was clearly evident, as demonstrated by fluorescence (green) staining in the blood vessels walls of animals 6 months of age (Figure 3). In contrast, blood vessels from animals 24 months of age show little to no detectable staining for MnSOD. Quantification of MnSOD staining (Figure 3) confirmed a significant (p < 0.001) decrease in enzyme expression in vessels from old animals compared to vessels of young animals. These results were further confirmed by western blot (Figure 4). Expression of MnSOD examined in microvessels from rats 6, 12, 18, and 24 months of age showed a decrease in MnSOD level that was significant (p < 0.01) at 18 months of age (Figure 4).

### Brain vascular expression of inflammatory proteins decreased with age

**Figure 5:**
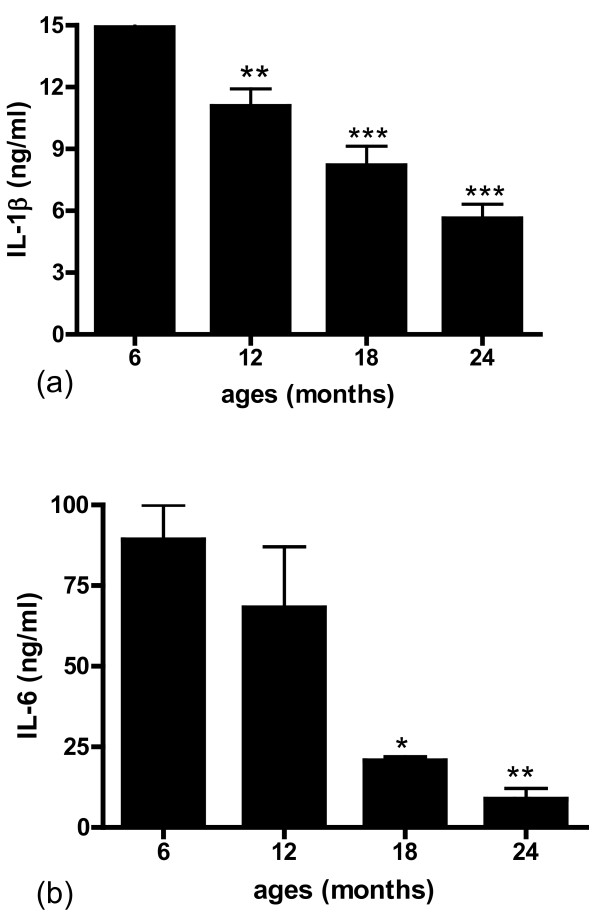
**Microvessels isolated from 6, 12, 18 and 24 month old rat brains were incubated for 8 h in serum-free media and the supernatant centrifuged (12,000 g, 15 min)**. IL-1β (5a) and IL-6 (5b) released into the supernatant were determined using rat custom 9 plex array ELISA (Pierce Biotechnology, MA). For IL-1β, ELISA was also performed using a kit from R<D Systems. Data are mean ± SD from 3 experiments performed in triplicate. * p < 0.05 vs. 6 months; **p < 0.01 vs. 6 months; ***p < 0.001 vs. 6 months.

We examined the ability of brain microvessels isolated from rats of different ages (6, 12, 18, 24 months) to release the cytokines IL-1β and IL-6. Brain microvessels isolated from these animals were incubated in serum-free-media for 8 h and the media collected and assayed by ELISA for IL-1β and IL-6. The amount of vascular-derived IL-1β showed a progressive decrease from 6 months to 24 months of age. This decrease was significant at 12 months (p < 0.01) and highly significant at 18 and 24 months of age (p < 0.001) (Figure 5a). Similarly, release of IL-6 showed an age-dependent decrease, with large (90%) and significant (p < 0.01) decrease at 24 months of age (Figure 5b).

**Figure 6:**
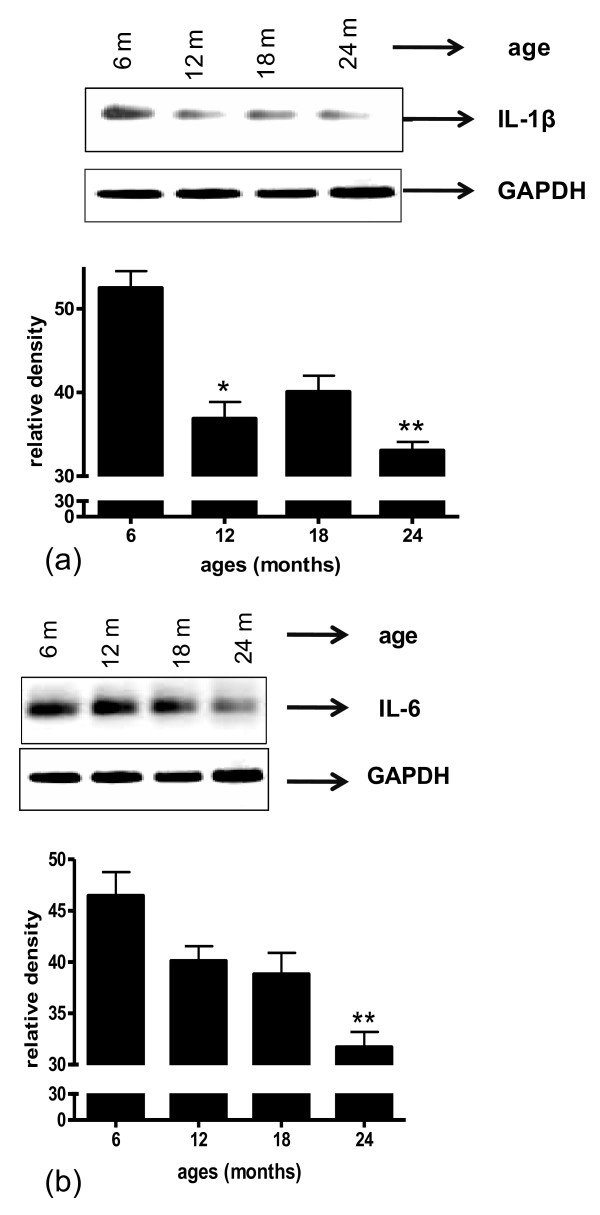
**Total RNA extracted from brain microvessels of 6, 12, 18 and 24 month old rats was reverse transcribed and amplified with (a) IL-1β or (b) IL-6 specific primers**. Amplified products were visualized in 1.5% agarose gel and band intensity represented as bars. Data were normalized relative to GAPDH expression. Data are mean ± SD from 3 experiments performed in triplicate. *p < 0.05 vs. 6 months; **p < 0.01 vs. 6 months.

An examination of cytokine expression at the RNA level confirmed an age-dependent decrease in mRNA for IL-1β (Figure 6a) and IL-6 (Figure 6b) when compared to the expression of the housekeeping gene GAPDH.

**Figure 7:**
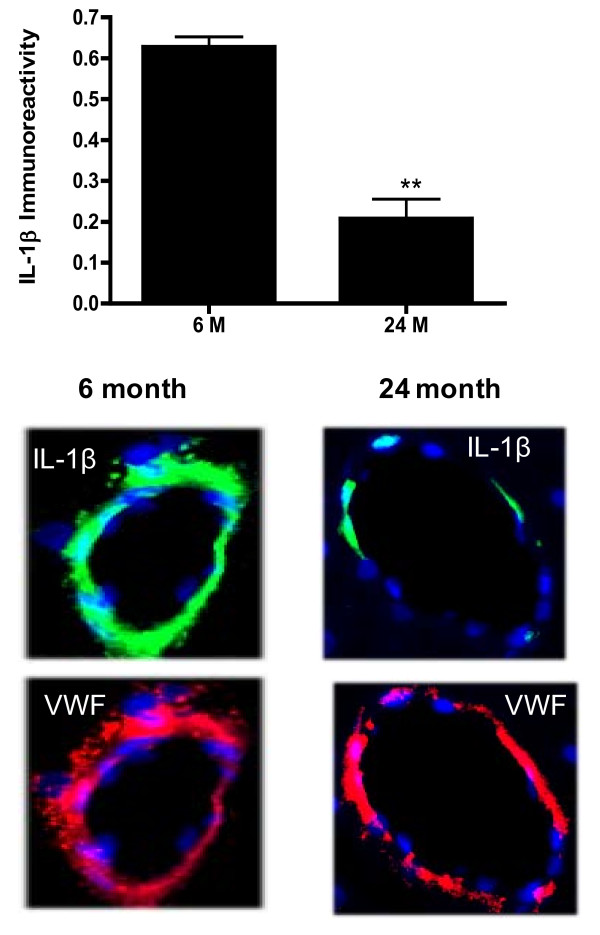
**Brain tissue sections from 6 and 24 month old Fisher 344 rats were stained for nuclear stain DAPI (blue) and either IL-1β (green) or the endothelial cell specific marker VWF (red)**. Quantitative comparison of IL-1β expression between vessels derived from 6 month and 24 month old animals is shown in bar graph. Data are mean ± SD (n = 3). ×20. **p < 0.01 vs. 6 months.

**Figure 8:**
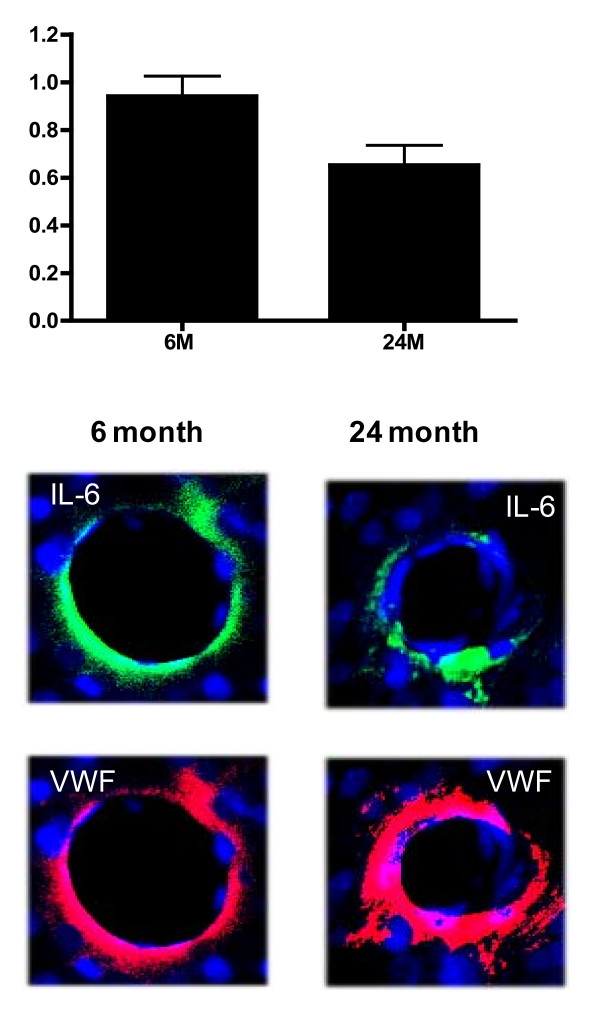
**Brain tissue sections from 6 and 24 month old Fisher 344 rats were stained for nuclear stain DAPI (blue) and either IL-6 (green) or the endothelial cell specific marker VWF (red)**. Quantitative comparison of IL-6 expression between vessels derived from 6 month and 24 month old animals is shown in bar graph. Data are mean ± SD (n = 3). ×20. *p < 0.05 vs. 6 months.

To further confirm these data, we examined the expression of IL-1β and IL-6 protein in tissue sections using immunofluorescent staining. The results indicated that reactivity to the antibody for IL-1β was intense in blood vessels from rats 6 months of age but significantly (p < 0.01) less (64.2%) in blood vessels from rats 24 months of age (Figure 7). Results obtained with antibody to IL-6 also indicated intense green fluorescent labeling in blood vessels from young rats (6 months of age), whereas staining was significantly (p < 0.05) lower in vessel walls from animals 24 months of age (Figure 8).

An examination of microvessel-conditioned media from rats 6, 12, 18 and 24 months of age for levels of inflammatory proteins IL-1α, MCP-1, TNFα, and IFN-γ showed a significant (p < 0.05-0.01) decline in these proteins as animals aged (Table 2).

**Table 2:** Effect of age on inflammatory proteins released by brain microvessels

Proteins (ng/ml)	6 month	12 month	18 month	24 month
IL-1α	1.87 ± 0.31	1.53 ± 0.23	1.13 ± 0.5	0.47 ± 0.12**

MCP-1	12.27 ± 1.22	7.9 ± 5.11	2.97 ± 1.87*	2.07 ± 1.21*

TNFα	7.40 ± 1.91	3.20 ± 1.73*	2.20 ± 0.0**	1.87 ± 0.58**

IFNγ	21.6 ± 1.40	19.67 ± 1.47	14.43 ± 1.78	8.87 ± 5.99**

Microvessels isolated from 6, 12, 18 and 24 month old rat brains were incubated for 8 h in serum-free media and the supernatant centrifuged (12,000 g, 15 min). Proteins released into the supernatant were determined using rat custom 9 plex array ELISA (Pierce Biotechnology, MA). Data are mean ± SD from 3 experiments performed in triplicate. *p < 0.05 vs. 6 month; **p < 0.01 vs. 6 month

## Discussion

Results from the current study indicate that expression and release of cytokines IL-1β and IL-6 are reduced in brain blood vessels with increased age. Release of IL-1α, MCP-1, TNFα, and IFN-γ from brain microvessels also decreases with age. Futhermore, we document that antioxidant protein expression decreases while oxidative stress and release of neurotoxic factors increase in the cerebromicrovasculature with age.

There are conflicting data as to whether indices of inflammation, such as cytokine production, increase, decrease or remain unchanged as humans or animals age [7,26,27]. Some reports show an increase in levels of circulating inflammatory proteins in the blood, including TNFα and IL-6, as well as a positive correlation between age and levels of IL-1β in isolated human monocyte cultures stimulated with LPS [3-6,27]. On the other hand, increasing age is associated with a decreased capacity of the immune system to mediate effective immune responses to vaccination and invading pathogens [28-31]. This age-related decrement in immune function is referred to as immunosenescence. Inflammation, a specialized immune response, might be expected to decline with age. In this regard, LPS-stimulated whole blood supernatants from elderly subjects exhibit lower TNFα and IL-1β levels compared to samples from young populations [9]. Differences in the health status of elderly subjects may, in part, explain contradicting results. Most human studies use volunteers that self-report health status. Thus, elevated IL-6, arguably a potential marker for a variety of diseases, could indicate concomitant inflammatory disease and/or poor nutritional status and indeed has been shown to be elevated in the elderly where C-reactive protein (CRP), an indicator of inflammation, is also high [13]. In this regard, a study where subjects were rigorously screened for good health found age did not influence basal secretion of IL-6 from peripheral blood monocytes (PBMCs), and that stimulated PBMCs from old patients produce less IL-6 than samples from young [7].

Experimental studies in animals that show contradictory alterations in inflammatory mediator expression and release with aging may reflect differences in tissue source and/or cell type. Results of the current study are focused exclusively on brain vascular changes. Comparing expression of IL-1β and IL-6 between young and old rats we document the decrease in cytokine expression at the RNA level and protein level, using RT-PCR, ELISA and immuno-histochemistry. At the protein level a decrease in IL-1β and IL-6 is demonstrable both by a rat custom 9 plex assay performed by Pierce Biotechnology, and confirmed in our laboratory by ELISA. Although immunohistochemistry is not a quantitative technique, expression of inflammatory proteins is pronounced in the vasculature in brain tissue sections from young animals and is greatly reduced in samples from old rats. These data support the idea that vascular-derived IL-1β and IL-6 decrease with age.

Whether specific cytokines are a trophic or toxic influence in the brain is unclear. The cytokines IL-1β and IL-6 are produced within the CNS, and similar to the periphery, they exhibit pleiotropic and contradictory functions. IL-1β has been identified as a mediator of several forms of neurodegeneration in the brain. Intracerebral administration of IL-1β results in acute brain injury characterized by apoptotic cell death and elevated expression of amyloid beta precursor protein [32]. In rat glial-neuronal cultures IL-1β induces neuronal cell death, via astrocytic release of matrix metalloproteinase-9 [33]. In contrast, cultured neurons treated with IL-1β are protected against the neurotoxic effects of NMDA-mediated injury; an effect dependent on IL-1β-mediated release of nerve growth factor [34]. Also, the protective effect afforded by ischemic preconditioning is associated with an increase in IL-β, indicating the importance of timing [35]. In a similar manner, IL-6 can produce either deleterious or beneficial effects on neuronal function. IL-6 protects neurons against glutamate- and NMDA excitotoxicity *in vitro *and prevents brain from ischemic or excitotoxic attacks *in vivo *[34,36]. However, other studies have implicated IL-6 in the pathogenesis of neurodegenerative disorders [37]. Finally, experiments using neuronal-astrocytic cultures show that IL-6 is both neuroprotective and neurotoxic, depending on concentration [38]. Taken together, data highlight the importance of context in determining functional significance of inflammatory protein expression.

A consistent literature indicates that oxidative stress is a feature of neurodegenerative diseases and aging [reviewed in [39]]. The oxidative stress-free radical theory of aging proposes that endogenously produced oxygen radicals are a basic cause of progressive age-associated declines in tissue function [40]. In addition, there are complex interactions between inflammatory proteins and oxidative stress. For example, IL-6 has been shown to protect PC12 neuronal cells from 4-hydroxynonenal-induced cytotoxicity by an increase in intracellular glutathione levels [41]. Addition of an antioxidant grape seed extract improves survival of cultured neurons exposed to H_2_O_2 _via release of IL-6 [42]. In the current study we document a progressive increase in vascular carbonyl content with increasing age. Elevated protein carbonyl formation, the most widely studied marker of protein oxidation, has been documented in both normal aging as well as in AD [39,43]. Also, oxidative stress reflects an imbalance between reactive species generation and the ability of antioxidant systems to buffer these radicals. An age-related attenuation of microvascular antioxidant defenses has been suggested [44]. Our results show that reactivity to the MnSOD antibody is robust in vessels from young animals, but is barely detectable in vessels of aged Fischer 344 rats. This decrease in the mitochondrial antioxidant MnSOD may be especially relevant for the cerebral microcirculation because endothelial cells have an extensive mitochondrial network [45]. Mitochondria are both sources of and targets for reactive oxygen species, and there is growing evidence that mitochondrial dysfunction may be an important mediator of vascular lesions [46,47]. The brain vasculature appears especially sensitive to oxidative stress. This sensitivity may in part be due to higher levels of NAD(P)H-oxidase in brain endothelial cells compared to endothelial cells in peripheral vessels. In this regard, a recent study shows that the inflammatory protein CRP evokes NAD(P)H-oxidase dependent functional derangements in brain- but not aorta-derived endothelial cells [48]. Our results, showing a decrease in MnSOD and increase in vascular dysfunction, as evidenced by release of neurotoxins, are supported by studies that link decreased MnSOD with increased endothelial dysfunction [49,50].

Dynamic communication between the cells of the neurovascular unit is required for normal brain functioning [51]. Endothelial cells are a key component of the neurovascular unit and are highly synthetic cells. Activated endothelial cells elaborate adhesion molecules, cytokines, chemokines growth factors, vasoactive molecules, procoagulant and anticoagulants moieties and variety of other gene products with biologic activity. The activated endothelium exerts direct local effects by producing paracrine factors that act on adjacent cells [52]. Pathologically activated or dysfunctional endothelial cells are key to the development of the age-associated cardiovascular disease atherosclerosis and are increasingly implicated in the development of disorders of the CNS [53]. We have shown synthesis and release of neurotoxic thrombin from the cerebro-microcirculation in AD [25]. In the current study we document that aging, in the absence of disease, causes changes in brain blood vessels that result in release of factors that are injurious to neurons. These data highlight a role for vascular-derived products as contributing to a noxious microenvironement for neurons in the aging brain. Understanding how aging affects vascular expression of inflammatory proteins, oxidative stress markers, and neurotoxins could provide insight into how aging predisposes the brain to development of age-related diseases and suggest strategies to improve brain function in aging.

## Authors' contributions

DT carried out the protein carbonyl studies, the ELISA and RT-PCR studies, the western blot analysis, participated in the microvessel isolation and cell culture procedures, ran statistical analysis on the gathered data and created the initial manuscript draft. XY performed the immunofluorescence studies and analysis. AS performed the microvessel isolation and cell culture procedures. JL participated in the design and coordination of the study as well as revised the manuscript for intellectual content. JM aided in the drafting of the manuscript and participated in the revision of the manuscript for intellectual content as well as the interpretation of data. PG is the laboratory PI, participated in the design and coordination of the study, aided in drafting the manuscript, revised the manuscript for intellectual content, interpreted data and gave final approval of the manuscript. All authors read and approved the final manuscript.

## Competing interests

All authors have contributed to the work and agree with the presented findings. This work has not been published before nor is it being considered for publication by another journal. There is no conflict of interest with any of the authors.
